# 新型无水胸腔负压引流装置在胸外科术后患者中的应用研究

**DOI:** 10.3779/j.issn.1009-3419.2020.104.06

**Published:** 2020-06-20

**Authors:** 文峰 俞, 亮 潘, 洁萍 张, 芃 叶, 政良 屠, 望 吕, 坚 胡

**Affiliations:** 310003 杭州，浙江大学医学院附属第一医院胸外科 Department of Thoracic Surgery, the First Affiliated Hospital, Zhejiang University School of Medicine, Hangzhou 310003, China

**Keywords:** 无水, 负压, 闭式引流, Anhydrous, Negative pressure, Closed drainage

## Abstract

**背景与目的:**

胸外科术后常规会放置胸管行闭式引流排出胸腔内积气、积液促进肺复张，并且可以观察术后是否有活动性出血，是否有肺漏气现象。常规胸腔闭式引流外接水封瓶，通过观察引流情况、水柱波动来判断病情，是非常经典的方法。但是水封瓶有易倾覆、携带不便等缺点，不利于患者早期活动。在加速康复的理念下，本中心应用了新型无水胸腔负压引流装置，并取得了较好的效果。本研究旨在观察新型无水胸腔负压引流装置在胸外科术后患者中的应用效果。

**方法:**

回顾性分析2018年1月-2019年12月在浙江大学医学院附属第一医院胸外科行肺部手术的患者，分成两组，一组患者使用传统胸腔闭式引流水封瓶作为对照组，另一组使用新型无水负压引流瓶作为实验组。统计患者的性别、年龄、高血压、糖尿病、吸烟史、手术切口和手术方式以及患者在院时间和术后住院时间。

**结果:**

两组患者在年龄、性别、合并症（高血压、糖尿病、吸烟史）、手术范围、手术时间方面并无统计学差异，但是两组患者在手术使用切口方面存在着统计学差异（*P*=0.01）。使用新型无水负压引流器的患者无论在术后住院时间以及总住院时间方面都比有水负压引流器的患者短，差异有明显统计学意义（*P*=0.02, *P*=0.04）。

**结论:**

新型无水胸腔负压引流装置对于胸外科术后快速康复推进有较好的作用。

## Abstract (en)

**Background and objective:**

After general thoracic surgery, a chest tube is usually placed for closed drainage to expel gas accumulation in the thoracic cavity and fluid accumulation to promote lung re-expansion. It can also be observed whether there is active bleeding after the operation and whether there is a pulmonary leak. The conventional drainage of the chest cavity is connected with a water-sealed drainage bottle, and the patient condition is judged by observing the drainage situation and the fluctuation of the water column, which is a very classic method. However, the water-sealed bottle has the disadvantages of being easy to overturn and inconvenient to carry, which is not conducive to the early activities of patients. Under the concept of accelerated rehabilitation, our center applied a new type of anhydrous thorax negative pressure drainage device and achieved good results. The purpose of this study was to observe the effect of a new type of anhydrous thoracic negative pressure drainage device in patients after thoracic surgery.

**Methods:**

Retrospective analysis of patients who underwent lung surgery in the First Affiliated Hospital of Zhejiang University Medical College from January 2018 to December 2019, patients were divided into two groups. One group of patients used a traditional closed-chest drainage water-sealed bottle as a control group, and the other group used a new type of anhydrous negative-pressure drainage bottle as an experimental group. Patients' gender, age, hypertension, diabetes, smoking history, surgical incisions and surgical methods, and the length of hospital stay and postoperative hospital stay were calculated.

**Results:**

There were no statistical differences in age, gender, comorbidities (hypertension, diabetes, smoking history), scope of surgery, and duration of surgery between the two groups of patients, but there were statistical differences in surgical incisions between the two groups of patients (*P*=0.01). We found that patients using the new waterless negative pressure drainage device were shorter than patients with water negative pressure drainage device in terms of postoperative hospital stay and total hospitalization time, and the difference was statistically significant (*P*=0.02, *P*=0.04).

**Conclusion:**

The new type of anhydrous thoracic negative pressure drainage device has a good effect on the rapid recovery and advancement after thoracic surgery.

肺部手术患者术后胸腔积气、积液的顺利引流是影响患者术后康复的重要因素。手术中麻醉、肺部创伤、肺功能等因素会增加胸腔积液以及肺不张、肺感染的可能性，因此肺部手术患者术后都会常规行胸腔闭式引流术^[1]^。传统的胸腔闭式引流管外接的是水封瓶，瓶体笨重携带不便，患者易牵拉疼痛，给患者术后康复训练造成诸多不便，对于胸腔术后患者的快速康复是不利的^[2]^。鉴于此，本研究拟评价一种新型无水负压引流瓶与传统胸腔闭式引流水封瓶对肺部手术患者术后快速康复的作用。

## 1 资料与方法

### 1.1 临床资料

回顾性分析2018年1月-2019年12月在浙江大学医学院附属第一医院胸外科行肺部手术的患者，分成两组，一组患者使用传统胸腔闭式引流水封瓶作为对照组，另一组使用新型无水负压引流瓶作为实验组。统计患者的性别、年龄、高血压、糖尿病、吸烟史、手术切口和手术方式以及患者在院时间和术后住院时间。纳入标准：①肺部无创伤病史；②术前未进行放疗；③非小细胞肺癌患者；④进行肺癌手术的患者。排除标准：①合并其他肿瘤；②小细胞肺癌患者；③进行纵隔和食管手术的患者；④临床资料不全患者。

### 1.2 新型引流器使用

本次研究采用的是浙江祥康医疗科技有限公司生产的新型无水负压引流瓶（图 1）。产品有A和B两种型号。A型抽吸器初始负压值≤-20 kPa，B型抽吸器初始负压值≤-20 kPa。本次使用的是A型。产品由抽吸器、悬挂装置、引流管、罗伯特夹、接头、盖体、塑片单向阀、集液袋、阻水排气装置组成。性能方面：使用时向引流器内冲入50 cm水柱的压力，应无气体泄漏；舌瓣单向阀开启压应≤2 cmH_2_O，阻水排气装置其阻水压力值≥10 kPa，其排气开启压力值应≤0.5 kPa，引流器1, 000 mm高度降落到硬质平面上，仍能保持其供使用状态；向引流器集液袋施加500 N垂直压力，引流器仍无泄漏；产品应经已确认过的消毒过程：以枯草杆菌黑色变种芽袍为指示菌。使用时（1）在消毒有效期内拆封包装后，关闭罗伯特夹，将接头与预埋在引流部位的引流导管*连接，打开罗伯特夹，将产品悬挂在低于引流部位的位置，观察应有液体流入引流接管内（*符号为医疗单位自备器械）。（2）双手平行挤压抽吸器，产品转为负压引流；在负压引流状态时，产品与术部位置平行线下的高低差及方位不受限制。（3）当风箱缓慢膨起复原时，产品仍在重力引流。（4）更换产品时，应在罗伯特夹处于关闭状态下进行。（5）在使用带放液开关产品时，应先将放液开关关闭，再执行上述步骤。

**1:**
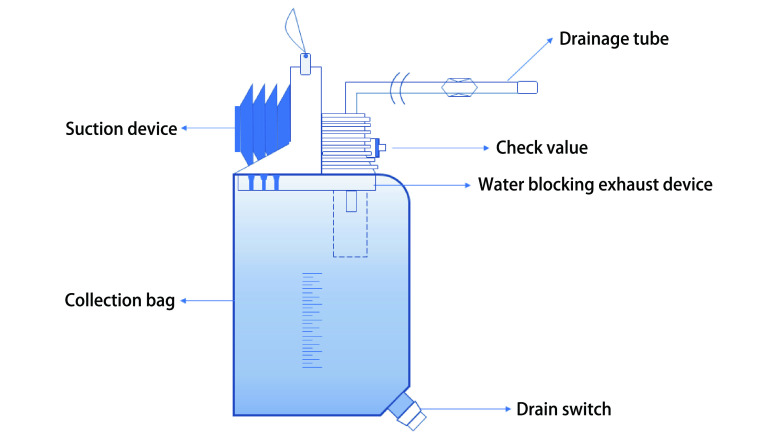
新型无水负压引流器示意图 New type of anhydrous thoracic negative pressure drainage device schematic diagram

### 1.3 手术过程使用方法和统计指标

手术方式为胸外科常规术式。包括单孔胸腔镜、三孔胸腔镜及达芬奇机器人辅助腔镜。术式包括肺楔形切除术、肺段切除术及肺叶切除术。术后患者常规使用水封瓶行闭式引流，观察患者是否存在漏气，如无漏气，于术后第二天更换负压无水引流袋。统计指标包括患者年龄、性别、基础情况、切口方式、手术方式、手术时长及住院天数。

### 1.4 统计学方法

采用SPSS 19.0进行统计学分析，计量资料采用均数±标准差（Mean±SD）表示，计数资料采用百分比表示，计量资料的组件比较采用独立样本*t*检验，计数资料的组间比较采用卡方检验，*P* < 0.05为有统计学差异。

## 2 结果

### 2.1 患者临床资料

我们先对使用有水负压引流器的423例患者和使用新型无水负压引流器的69例患者的基线资料进行分析，结果如表 1所示。我们发现两组患者在年龄、性别、合并症（高血压、糖尿病、吸烟史）、手术范围、手术时间方面并没有存在统计学差异，但是两组患者对于手术使用切口方面存在着统计学差异（*P*=0.01）。同时，我们将关注两组患者在术后使用不同的胸腔引流器之后在术后住院时间以及总住院时间方面存在的差异。我们发现使用新型无水负压引流器的患者无论在术后住院时间以及总住院时间方面都比有水负压引流器的患者短，而且差异具有明显的统计学意义（*P*=0.02, *P*=0.04）。

**1:** 患者临床资料 Clinical characteristics of patients

	General drainage group (*n*=423)	Anhydrous negative pressure drainage group (*n*=69)	*P*
Age (yr)	60.1±10.9	58.4±10.9	0.83
Gender			0.36
Male	211 (49.9%)	30 (43.5%)	
Female	212 (50.1%)	39 (56.5%)	
Comorbidities			
Hypertension	129 (30.5%)	19 (27.5%)	0.67
Diabetes	42 (9.9%)	4 (5.8%)	0.37
Smoking	136 (32.2%)	14 (20.3%)	0.05
Incision			0.01
Uni-portal	48 (11.3%)	19 (27.5%)	
Double-hole	12 (2.8%)	1 (1.4%)	
Three-hole	274 (64.8%)	38 (55.1%)	
Four-hole	37 (8.7%)	8 (11.6%)	
Traditionalposterolateral incision	52 (12.3%)	3 (4.3%)	
Scope of surgery			0.28
Lobectomy	270 (63.8%)	39 (56.5%)	
Sublobectomy	153 (36.2%)	30 (43.5%)	
Duration of operation (min)	126.2±51.0	100.3±49.7	0.67
Postoperative hospital stay (d)	5.6±2.31	4.3±1.4	0.02
Total hospital stay (d)	13.2±4.4	10.5±3.2	0.04

### 2.2 两组术后特征

为了进一步了解不同手术切口对肺癌患者术后使用不同胸腔引流器的影响，我们选取了使用VATS辅助单孔切口进行肺癌手术的患者和使用VATS辅助三孔切口进行肺癌手术的患者进行术后住院时间以及总住院时间的比较。我们发现对于使用VATS辅助单孔切口进行肺癌手术的两组患者来说，无论是术后住院时间还是总住院时间方面，我们都没有发现有水负压引流组患者和无水负压引流组患者之间存在统计学差异（*P*=0.61, *P*=0.99）（表 2）。但是，当我们比较使用VATS辅助三孔切口进行肺癌手术的两组患者时，我们发现有水负压引流器的患者和使用新型无水负压引流器的患者术后住院时间方面存在统计学差异，且使用无水负压引流组患者比有水负压引流器的患者要短（*P*=0.01）（表 3）。

**2:** VATS辅助单孔切口进行肺癌手术的患者的术后特征（Mean±SD） Postoperative characteristics of patients underwent VATS assisted uni-portal incision(Mean±SD)

	VATS assisted uni-portal incision		*P*
	General drainage group (n=48)	Anhydrous negative pressure drainage group (n=19)	
Postoperative hospital stay (d)	4.9±1.6	3.8±1.4	0.61
Total hospital stay (d)	11.9±2.7	11.6±2.8	0.99

**3:** VATS辅助三孔切口进行肺癌手术的患者的术后特征（Mean±SD） Postoperative characteristics of patients underwent VATS assisted three-hole incision（Mean±SD）

	VATS assisted three-hole incision		*P*
	General drainage group (n=274)	Anhydrous negative pressure drainage group (n=38)	
Postoperative hospital stay (d)	5.4±2.1	4.2±1.1	0.01
Total hospital stay (d)	12.9±3.9	9.9±3.4	0.12

## 3 讨论

常规胸腔闭式引流瓶已在临床使用多年，具有一定的优势，比如产品价格经济。二十多年临床使用经验，经受住市场考验。少数高端胸腔闭式引流瓶昂贵，功能相同。临床习惯水柱波动观察。水封式引流仍是最直观的引流方式。气胸患者使用水封瓶可较直观反映漏气程度。但是常规胸腔闭式引流瓶也具有一定缺点，比如产品体积庞大、瓶体重、不易携带。产品易打翻，破损产品非预期使用，引流管易脱落。如需实现负压吸引功能，只能采用三瓶结构的胸腔闭式引流瓶，躺在病床上接中央吸引器进行负压引流^[3]^。因产品吹塑工艺结构的原因，瓶子在运输过程中遇到野蛮作业时，有造成产品破损的风险，瓶体等同于胸腔，如有破损未及时发现，极易造成开放性气胸等医疗事故。产品操作较繁琐，拧开瓶盖，加生理盐水至刻度线，关闭瓶盖，连接连通管与胸腔引流导管后方可使用。水封瓶较为笨重，患者术后易牵拉，造成疼痛及非计划性拔管^[4]^。而常规负压引流袋产品价格不等，材料目前PVC、硅胶等皆有，以适应临床多种引流需求。低-中-高压引流，引流环境多种。术后引流适用于全科室使用。常规负压引流袋（器）缺点是产品结构简单，功能单一，压力范围小，多无负压调节和补偿功能，如弹簧式引流器，压力调节范围小，抗返流装置简单，调节压力时需打开释放开关，挤压弹簧，然后闭合开关，此过程易造成医患人员的交叉感染，且无法杜绝返流现象。负压引流球引流容积小，需频繁挤压引流球，且返流现象普遍。临床无较好的选择方案^[5-7]^。

胸腔术后引流一直在不断地进行改进，胸管的管理一直是胸外科同道尚有争议的话题。近年来诸如数字引流系统对于肺漏气量的监测应用，负压吸引与水封引流的转换等。在快速康复的大背景下，胸管的管理显得尤为的重要^[8, 9]^。

本次使用的新型无水引流袋袋体强度及安全性高。患者可下床活动，康复锻炼时，换上该产品，产品便于携带，且结构稳定安全。可持续补偿并调节负压大小。轻松产生负压，挤压抽吸风箱来调节补偿负压。引流持续且彻底。双重超灵敏防逆流单向阀装置，杜绝逆行性感染。阻菌阻水排气装置为瑞典进口膜材料，可达到超强透气阻水阻菌。产品不受引流位置高低影响。产品随意倾倒且位置高低不影响引流效果，配合度差的幼儿亦可安全使用。无需加水封液，免除频繁挤捏导管。减少护理工作量，操作方便快捷，免除频繁挤捏导管的繁重护理。携带方便，提早下床，快速康复，提早出院。提高医院病床周转率，减轻患者负担。围手术期心理康复的护理。传统模式需携带大瓶小瓶一大堆，患者受异样眼光，身心负担较重，不愿积极参与活动。新模式下可将产品悬挂并隐藏于衣服内，维护病患隐私及尊严。

通过本次研究我们发现，对于多切口的患者，使用这种新型无水引流袋袋体相对于传统的有水引流装置，显示出加速患者康复的趋势。无水引流开创了胸外科引流的新纪元，由水封瓶到无水引流装置的转换是胸外科加速康复理念的转换^[10, 11]^。不可否认，水封瓶是极其经典的引流方式，已经有百余年的应用历史，但其局限性也一直未能打破。应用新型的无水负压引流装置使得患者的活动不再受限，不用担心水封瓶的倾覆，也不用限制其体位的变化，便携且安全，保持负压无逆流风险^[12, 13]^。

当然，我们也注意到，本次纳入研究的样本量较少，入组患者的统计变量较少，结果的可靠性还需要进一步的实验研究来证实。因此，我们需要进行更大规模的临床前瞻性试验来进一步研究新型无水引流装置对于患者的可靠性和安全性。
